# Inverted organic photovoltaic device with a new electron transport layer

**DOI:** 10.1186/1556-276X-9-150

**Published:** 2014-03-27

**Authors:** Hyeong Pil Kim, Abd Rashid bin Mohd Yusoff, Hyo Min Kim, Hee Jae Lee, Gi Jun Seo, Jin Jang

**Affiliations:** 1 Advanced Display Research Center, Department of Information Display, Kyung Hee University, Dongdaemoon-gu 130-701, Seoul, South Korea

**Keywords:** Lithium, Zinc oxide, Organic photovoltaic, Stability, Solution-processed, Electron transport layer, Blend ratio

## Abstract

We demonstrate that there is a new solution-processed electron transport layer, lithium-doped zinc oxide (LZO), with high-performance inverted organic photovoltaic device. The device exhibits a fill factor of 68.58%, an open circuit voltage of 0.86 V, a short-circuit current density of −9.35 cm/mA^2^ along with 5.49% power conversion efficiency. In addition, we studied the performance of blend ratio dependence on inverted organic photovoltaics. Our device also demonstrates a long stability shelf life over 4 weeks in air.

## Background

Next-generation organic photovoltaics (OPVs) require not only semiconductors with high performance, but also for them to feature high optical transparency, low temperature, and solution processability. Currently, metal oxide interlayers are widely used as hole and electron buffer layers in OPVs. However, OPVs have relatively low energy efficiency (12%) [1], and their deposition requires a costly vacuum deposition technique. Accordingly, in a search for an alternative for metal oxide interlayer, considerable attention has been given to metal oxide semiconductor, such as indium zinc oxide (IZO) [1-5], zinc oxide (ZnO) [6-10], aluminum-doped zinc oxide (AZO) [11-15], titanium oxide (TiO_*x*_) [16-20], graphene oxide (GO) [21-25], tungsten oxide (WO_*x*_) [26-30], nickel oxide (NiO_*x*_) [31-35], molybdenum oxide (MoO_*x*_) [36-40], vanadium pentoxide (V_2_O_5_) [41-45], and reduced graphene oxide (rGO) [46-50], as these oxides have high optical transparencies, excellent electrical properties, chemical stability, and solution processability. For instance, TiO_*x*_-based semiconductors have been successfully employed in various electronic devices, namely, as an electron transport layer for solar cells, energy harvesting devices with piezoelectric properties, and channel materials for thin film transistor (TFT). In OPVs, TiO_*x*_[20-25] films are deposited using various techniques, such as chemical bath deposition, spray pyrolysis, and rf magnetron sputtering. Although sputtering and vapor deposition methods have ensured high device performance and reliability, they are costly and the procedure is cumbersome. Nonetheless, solution processing of TiO_*x*_ allows large-area and low-cost manufacturing. However, it compromises the resulting energy efficiency as well as carrier mobility.

Thus, various zinc oxides such as IZO [6-10] which has good solution processability at relatively low temperature have been introduced. However, in spite of their good performance, we are now running out of indium supply. The price of indium has increased in the last 10 years, and indium has become increasingly scarce. Therefore, to resolve these problems, indium-free ZnO-based semiconductor has been introduced. Because these methods were based on a sol-gel process with hydrocarbon complexes, they all suffered from residual carbon impurities which hamper electron transfer. In order to remove the impurities, an annealing process must take place at a high temperature of at least 400°C. Furthermore, to improve the energy efficiency, the ZnO solution must be processed without the presence of indium compounds and carbon and must be processed at a low temperature. Recently, it is been reported that the carbon-free method at a low temperature can be processed and is compatible with mechanically flexible substrates. However, the electron mobility of ZnO as described in this study is inadequate for use in high-quality OPVs [51].

To overcome the aforementioned challenges, we adopted alkali metal dopants focusing particularly on lithium in order to fabricate solution-processed OPVs with enhanced energy efficiency and stability. We used lithium as a dopant because it has comparable conductivity (2.25 × 10^−6^ S/cm) compared to ZnO. According to literature, the cost of 100 g of bulk lithium is nearly US$10 [52]. We introduced a unique aqueous PEDOT:PSS-doped carbon nanotube (CNT), which successfully created a new interface engineering as the buffer layer at maximum process temperature as low as 80°C for 30 min. Additionally, the influence of the dopant concentration on the device performance was also examined. Lithium-doped zinc oxide (LZO) OPVs demonstrated a maximum energy conversion efficiency of 5.49%, with a fill factor of 68.58%. Furthermore, LZO devices also exhibited excellent shelf life in an air test that lasted over 30 days.

## Methods

### LZO solution preparation

Zinc acetate dihydrate (99.9%, Merck, Gangnam-gu, Seoul, South Korea) was used as a starting material and was recrystallized twice from double distilled water. The complexes with monoethanolamine have been prepared in ethanol solution. Resultant complexes of 1:1 stoichiometric composition have been obtained [4]. Lithium chloride was used as a doping agent in zinc-acetate-based precursors.

### Device fabrication

Patterned indium tin oxide (ITO)-coated glass with a sheet resistance of 15 Ω/sq was cleaned inside an ultrasonic bath. First, it was cleaned with deionized water, followed by acetone, methanol, 2-propanol, and finally with ultraviolet (UV) ozone. A ferroelectric solution-processed LZO solution was spin-coated (600 rpm) on the top of the ITO substrate and dried at 245°C for 10 min to form a thin interlayer of 35 nm. The thickness of the interlayer was determined following a previously published paper [4]. The active layer (P3HT:ICBA = 1:1, 1.5 wt.%), with a thickness of 85 nm, was then coated on top of the interlayer, by casting from a dichlorobenzene solution and drying it at 150°C for 10 min. The thickness of the active layer was verified by a surface profilometer (Alpha-Step profilometer; KLA Tencor, Milpitas, CA, USA). The PEDOT:PSS:CNT was coated on the active layer at 4,000 rpm for 25 s and later annealed at 120°C for 20 min. Finally, a 100-nm aluminum layer was evaporated with a shadow mask. The overlapping area between the cathode and the anode defined the device active area of 0.04 cm^2^. All the fabrication processes were carried out inside a controlled atmosphere of a nitrogen drybox (mBRAUN, Stratham, NH, USA) containing less than 10 ppm oxygen and moisture.

### J-V and EQE

The power conversion efficiencies of the inverted OPVs were measured under an illumination of AM1.5G simulated solar light (Oriel Model 91192; Oriel Instruments, Stratford, CT, USA) at 100 mW/cm^2^. The current density-voltage (*J*-*V*) characteristics were recorded with a Keithley 2410 source unit (Keithley Instruments Inc., Cleveland, OH, USA). The external quantum efficiency (EQE) measurements were performed using an EQE system (model 74000) obtained from Newport Oriel Instruments USA and Hamamatsu (Hamamatsu Photonics, Hamamatsu, Japan) calibrated silicon cell photodiode used as a reference diode. The wavelength was controlled with a monochromator at 200 to 1,600 nm.

### Ultraviolet photoelectron spectroscopy

Ultraviolet photoelectron spectroscopy (UPS) measurements were performed using the He I photon line (h*v* = 21.22 eV) of a He discharge lamp under UHV conditions (4 × 10^−10^ mbar). Emitted photoelectrons were collected using a semi-spherical channeltron with analyzer pass energy set to 5 eV. XPS was carried out using a MgKα line (h*v* = 1,253.6 eV) with an analyzer pass energy of 10 eV.

### Spectroscopy ellipsometry

Spectroscopy ellipsometry measurements were gathered using of a rotating analyzer Woollam variable angle spectroscopic ellipsometer (WVASE) system, equipped with a high-pressure Xe discharge lamp integrated in an HS-190 monochromator.

### Atomic force microscopy

Atomic force microcopy (AFM) imaging was completed in air using digital instrument multimode equipped with a nanoscope IIIa controller.

### Resistivity and Hall measurements

The measurement of Hall effect was carried out in Van der Pauw configuration with a constant current of 100 A using high-resolution instruments, with an accuracy of approximately 1.5%. Hall measurements were repeated for different magnetic fields and different applied voltages on the same sample.

## Results and discussion

**Figure 1:**
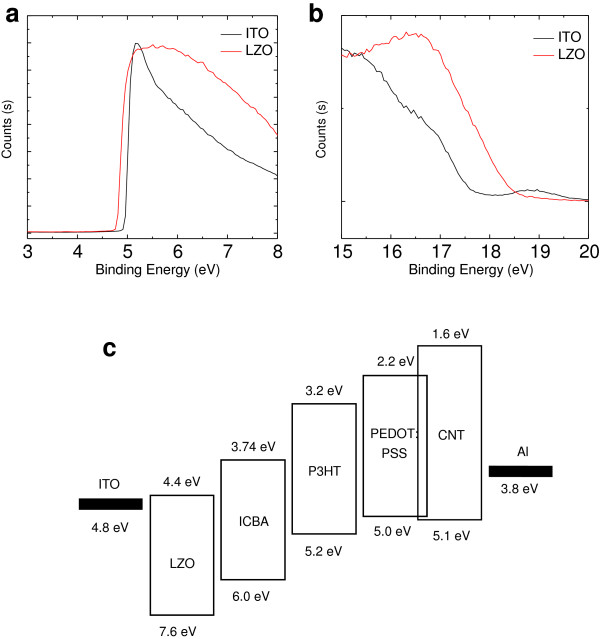
**UPS spectra of LZO. (a)** Inelastic cutoff region. **(b)** Fermi edge region. **(c)** Schematic energy levels and device structure are used in this study.

To explore the energy levels of LZO, we have performed UPS. Using UPS measurements, the work function values were calculated from the difference between the inelastic cutoff and the Fermi edge, as shown in Figure 1a,b. The ITO and LZO layers showed a work function of 4.7 eV and 4.6 eV, respectively.

The energy-level diagram illustrating the highest occupied molecular orbital and lowest unoccupied molecular orbital (HOMO-LUMO) of all materials used in the inverted OPVs, together with the work functions of the electrodes, is shown in Figure 1c. In this device, the LZO buffer layer may function as (i) electron-transporting and a hole/or exciton-blocking layer and (ii) as a modifier of the ITO work function at the ITO/P3HT interface, whereas the PEDOT:PSS:CNT buffer layer serves as a barrier layer by preventing the diffusion of the Al cluster (due to thermal evaporation) from penetrating through the photoactive layer.

As shown in Figure 1c, the LUMO of CNT (1.6 eV) is much higher than the LUMO of either donor P3HT (3.2 eV) or acceptor (3.74 eV), showing that CNT can efficiently block the backflow electron from indene-C60 bisadduct (ICBA) to Al electrode. This has shown good hole extraction ability in combination with Al as the top anode in inverted OPVs. LZO possesses conduction and valance bands of 4.4 and 7.6 eV, respectively, and can block backflow holes. Since the HOMO and LUMO levels of P3HT are 5.2 and 3.2 eV, respectively, the HOMO level of P3HT is well aligned to the HOMO level of CNT, which can benefit the hole extraction. The work function of LZO is 4.6 eV, which is moderately aligned with that of ICBA and can supply good electron extraction from the ICBA acceptor phase. Interestingly, photovoltaic results in this work indicate that PEDOT:PSS:CNT could efficiently extract holes from the P3HT to the Al anode. It is understood that the LZO interlayer can supply good electron extraction from the ICBA acceptor because of their moderately aligned conduction band. However, the LUMO level of 3.4 eV of ICBA is almost 0.9 eV higher than that of LZO. This is a moderate energy barrier for electron transport. According to the photovoltaic performances of the LZO-based inverted OPVs, electrons should be efficiently collected by the ITO cathode, suggesting that the LZO must afford a necessary electron pathway.

Hall effect measurement was carried out in Van der Pauw configuration for ZnO and LZO layers with different concentrations of Li. Some shallow donor levels in ZnO may give rise to n-type conduction. For ZnO:Li samples, Hall mobility and resistivity vary depending on the Li concentration as shown in Table 1. The results illustrate that the obvious trend of Hall mobility and resistivity decreases with increasing Li concentration. It is worth noting that impurity scattering reduces the mobility. Therefore, Hall mobility decreases at high Li concentration due to excessive impurity scattering. Thus, the resistivity of LZO films decreases when Li content reaches 30 wt.%, as shown in Table 1. The obtained results are in agreement with previously reported results for LZO thin film deposited at 500°C, giving rise to p-type conductivity along with hole concentration of about 1,016 cm^−3^[53,54]. The results clearly indicate that Li atoms substitute Zn in the Zn site to form shallow acceptor levels and p-type doping takes place in ZnO lattice. The increase in Madelung energy has been thought to facilitate the localization of such acceptor states [55].

**Table 1:** Summarized key parameters of inverted organic photovoltaics with different LZO concentrations

**LZO**	***J***_**sc**_	***V***_**oc**_	**FF**	**PCE**	***R***_**s**_	***R***_**sh**_	**Resistivity**	**Hall mobility**
**(%)**	**(mA/cm2)**	**(V)**	**(%)**	**(%)**	**(Ω cm**^**−2**^**)**	**(Ω cm**^**−2**^**)**	**(Ω cm)**	**(cm**^**2**^ **V s)**
0	−8.22	0.85	66.62	4.66	15.83	2,304	**-**	**-**
1	−9.19	0.86	65.04	5.15	17.71	2,159	**-**	**-**
2	−9.35	0.86	68.58	5.49	13.91	1,610	103.7	1.02
5	−9.15	0.86	65.21	5.12	16.62	1,598	-	-
10	−9.02	0.86	67.55	5.25	15.28	1,826	70.3	0.63
30	−8.73	0.86	65.92	4.95	17.07	2,222	20.3	0.21

*J*_sc_, short circuit current density; *V*_oc_, open circuit voltage; FF, fill factor; PCE, power conversion efficiency; *R*_s_, series resistance; *R*_sh_ shunt resistance.

**Figure 2:**
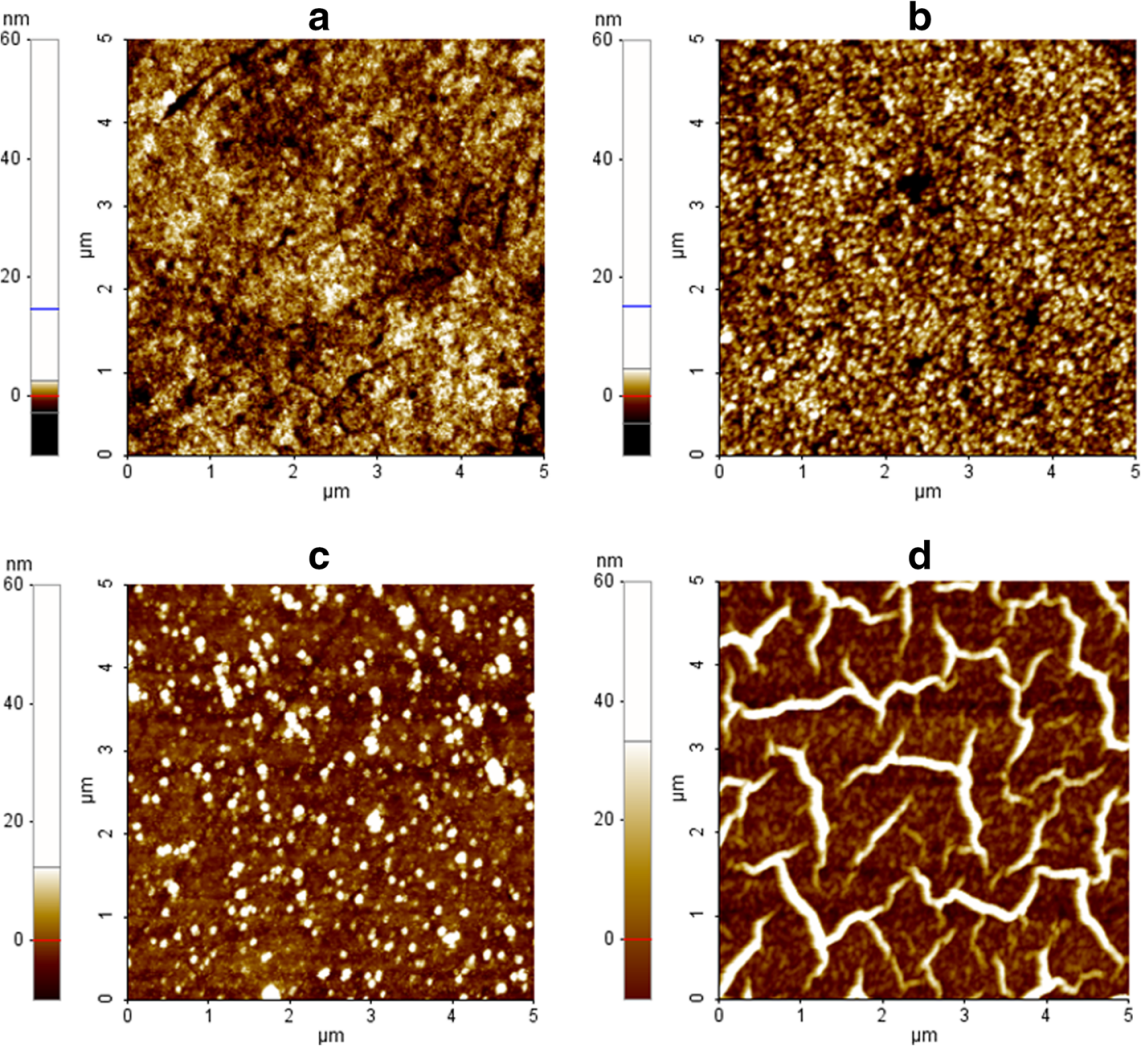
**AFM images. (a)** ZnO. **(b)** Li-doped ZnO (2 wt.%). **(c)** Li-doped ZnO (10 wt.%). **(d)** Li-doped ZnO (30 wt.%).

Figure 2 shows the AFM images of ZnO and LZO of 2, 10, and 30 wt.% Li-doped films, showing a variation with the increase of Li concentration. Figure 2b,c,d shows the AFM images of LZO (2, 10, and 30 wt.%) films showing formation of domains. The difference in domain morphology gives a clear indication of the decrease in magnetization from 2 to 30 wt.% Li doping in ZnO. Such domain formation indicates the occurrence of ferromagnetism. It can be said that intrinsic ferromagnetism in ZnO is possible with the doping of spin half-alkali atom Li. In addition, the surface of the ZnO thin film was very smooth, showing a low surface root-mean-square (RMS) roughness of 1.4 nm (Figure 2a). It was found that spin-coating the LZO 2 wt.% interlayer at 600 rpm for 25 s on the ITO substrate increased the roughness of the LZO layer (RMS = 2.3 nm, Figure 2b). Moreover, the 2 wt.% LZO morphology is uniform and dense. In the spin coating of LZO 10 wt.% (Figure 2c) interlayer, one finds that the RMS roughness increased abruptly (6.3 nm) with a lot of spikes over the entire surface. However, as the concentration of Li increases from 10 to 30 wt.%, the RMS roughness increases to 17 nm (Figure 2d) with poor surface morphology.

**Figure 3:**
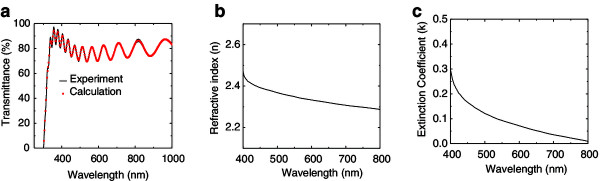
**Fitted transmittance spectrum, refractive index, and extinction coefficient of LZO layer. (a)** Fitted transmittance spectrum of LZO with 2 wt.% of Li. **(b)** Refractive index. **(c)** Extinction coefficient of LZO layer with 2 wt.% of Li.

Figure 3a shows the typical fitting plot of the transmittance spectrum for the ZnO thin film with 2 wt.% of Li. The derived value of the refractive index *n* (LZO 2 wt.%) is shown in Figure 3b, which has a comparable value to the ZnO single crystal measured by Yoshikawa and co-workers [56]. From this figure, the refractive index decreases with the increasing wavelength (400 to 800 nm). In addition, Figure 3c shows the extinction coefficient of the LZO as a function of wavelength. It can be seen that the extinction coefficient also decreases with the increased wavelength.

The effect of LZO on the performance of OPVs is as follows: the complete inverted OPVs which consist of ITO/cathode buffer layer/P3HT:ICBA/anode buffer layer/Al structure were fabricated to study the electron buffer layer dependence, the EQE trends, current-voltage characteristics under AM1.5G (100 mW/cm^2^) solar-illuminated conditions, and device stability over 720 h. The blend ratio of the cathode buffer layer was changed from 0 to 30 wt.% by controlling the concentration of lithium.

**Figure 4:**
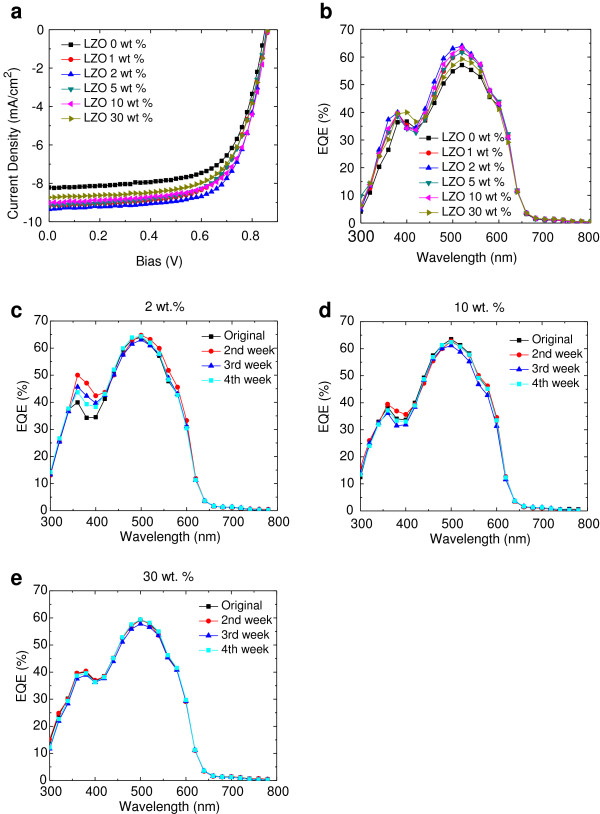
***J-V *characteristics of the prepared inverted OPVs.** Current density-voltage characteristics **(a)**. External quantum efficiency of inverted OPVs with LZO as a cathode buffer layer **(b)**. The evolution of EQE curves of LZO for three concentrations as a function of wavelength **(c)** 2 wt.%, **(d)** 10 wt.%, and **(e)** 30 wt.%.

The photocurrent *J*-*V* characteristics of the prepared inverted OPVs are shown in Figure 4. Short circuit current density (*J*_sc_), open circuit voltage (*V*_oc_), fill factor (FF), and power conversion efficiency (PCE) are summarized in Table 1. In this inverted device, the ITO work function is modified by introducing LZO film (UPS data - Figure 1a). Thus, the difference in work functions between top and bottom electrodes supports the hole transport from the ITO (cathode) through the multilayer of thin films towards the Al (anode) in short circuit conditions. Furthermore, LZO serves as a hole blocking layer in the environmentally stable and reliable inverted OPVs. This plays a vital role in obtaining an electron-selective contact at the ITO electrode. In this work, we have employed approximately 100 nm Al as the top electrode for better stability. The 2% LZO-based inverted OPVs displayed *V*_oc_, *J*_sc_, and FF values of 0.86 V, −9.35 mA/cm^2^, and 68.58%, respectively, giving a PCE of 5.49% (Figure 4 and Table 1). Improved photovoltaic performance is due to the higher *J*_sc_.

The large PCE improvement of the LZO 2 wt.%-based inverted OPVs is due to the largely elevated *J*_sc_ and FF. The average PCE of four LZO-2 wt.%-based inverted OPVs was 5.31%, demonstrating that LZO is a promising cathode buffer layer for high *J*_sc_ and high-efficiency OPVs. It should be noted that the *V*_oc_ of 0.86 V for LZO is the highest among all metal oxide electron extraction materials. Some metal oxides also demonstrate high *V*_oc_ in OPVs; however, these metal oxides showed lower PCE than that of LZO OPV. As the weight percentage increases to 10 and 30, the efficiencies of both devices decrease to 5.25 and 4.95, respectively. This indicates that Li doping enhances electron transport through ZnO. However, increasing the amount of Li led to a decrease in energy efficiency, which implies that Li doping of ZnO beyond 2 wt.% hampers electron transport. Such a decrease in *J*_sc_ and FF is caused when a significant amount of impurity (Li) is added to a ZnO film. This actually generates a number of charge traps. Additionally, when the *J*_sc_ is not saturated, it decreases when increasing the blend ratio. This can be seen in the case of a typical P3HT:ICBA bulk heterojunction inverted OPVs. When analyzing *J*-*V* characteristics, the amount of absorbed photon reduces depending on the blend ratio. Nevertheless, the amount of internal series resistance and deactivated carriers in the inverted OPVs actually increases, owing to the short carrier diffusion length in organic semiconducting materials. Due to the fact that LZO possesses low carrier mobility (Table 1), the transport process of the photogenerated carriers to the electrode could be inefficient. Consequently, we see that the *J*_sc_ is reduced even if high blending ratio is employed in our cells. However, FF decreased because of internal series resistance involving a cathode buffer layer in the LZO system. As we can see from *J*-*V* curves from Figure 4a, the blend ratio of 2 wt.% of the cathode buffer layer is the optimal value to achieve the best efficiency. We argue that the improvements on energy efficiency are possible because of two distinct reasons. The primary reason is that introductions of alkali metal (Li) will basically n-type dope ZnO and secondly for efficient hole collection and in-plane conductivity of our OPVs. A theory predicts that the presence of Li in the ZnO matrix prefers the interstitial sites (electron donor) over substitutional sites (acceptor) and thus n-type dopes ZnO. Shallow donors were experimentally tested in regard to Li atoms on ZnO. This was previously analyzed based on electron paramagnetic resonance and electron nuclear double resonance experiments [57]. Concordantly, we observed an increase in *V*_oc_, implying that adding Li beyond a certain amount creates a greater number of defects in the ZnO film rather than doping them. Because CNT is a mixture of p-doped nanotubes and metals, PEDOT:PSS is required to adjust the CNT work function for hole collection. At the same time, it must avoid any shorts in the device by having metallic nanotubes transport electrons from the ICBA network to the appropriate electrode. The charge transfer and transport processes can be understood from the energy level diagram depicted in Figure 1c. As discussed above, the *J*_sc_ is not saturated and decreases with the increase of the LZO blend ratio. In PEDOT:PSS:CNT-based devices, the inverted OPVs work at a higher blend ratio (30 wt.%). Although it seems like a higher blend ratio reduces the absorbed photon, the transport process in the PEDOT:PSS:CNT-based devices seems to be efficient. This is also evident because it is resistive of the inverted OPVs, where the series resistance of 2, 10, and 30 wt.% is 13.91, 15.28, 17.07 Ω cm^−2^, respectively. Series resistance (*R*_s_) and shunt resistance (*R*_sh_) values for the inverted OPVs are also listed in Table 1. The *R*_s_ of the (LZO 2 wt.%) PEDOT:PSS:CNT-based OPVs was the smallest, and the other two devices did not show too much *R*_s_ difference. The *R*_sh_ values of the PEDOT:PSS:CNT-based OPVs were obviously smaller.

The EQEs of inverted OPVs with LZO and PEDOT:PSS:CNT-based devices (Figure 4b) show a slightly lower EQE_max_ of 64% at 520 nm (LZO 2 wt.%). The EQE curve is similar to that of the LZO 10 and 30 wt.%-based OPVs. For LZO 10 and 30 wt.%, the EQE values are about 63.4% and 59.3%, respectively. The integration of the EQE curves for LZO 2, 10, and 30 wt.% is in agreement with the *J*_sc_ values obtained from *J*-*V* characteristics (9.30, 8.93, and 8.55 mA/cm^2^, respectively).

According to literature, ZnO-based inverted OPVs have demonstrated good results of shelf life in air [6-10]. In this study, in the shelf life test in air of inverted OPVs with LZO as the interlayer, two inverted OPVs with different anode buffer layers were compared. In order to protect the top Al electrodes of OPVs, we covered the active layer of each device with a glass plate with UV glue in a N_2_ glovebox. After 1 min of UV curing, the devices were placed in air under ambient conditions for stability testing. Therefore, we used PEDOT:PSS:CNT as the interlayer on active layer to construct an inverted OPVs. This provided a promising shelf life test in air. It may be expected that using a more stable metal (Ag or Au) to replace Al as the top anode in the inverted OPVs would improve the shelf life test in air.

The stability of the inverted OPVs was measured over a period of 30 days. *J*-*V* and EQE characteristics for PEDOT:PSS:CNT devices were recorded in the mentioned time interval. The photovoltaic parameters were derived as a function of the degradation time (expressed in week), as tabulated in Additional file 1: Table S1. The PEDOT:PSS:CNT devices (Figure 4c,d,e) demonstrate significant results in the shelf life test in air during the first 2 weeks. Later, results start to decrease. However, results are still higher by nearly 4% even after being in storage for 4 weeks. The increased efficiency of PEDOT:PSS:CNT devices is due to a slight increase of *J*_sc_ and FF values in time. This can be attributed to improved charge transport and electron collection phenomena. We can speculate that the solution-processed LZO film becomes more crystalline over time. Thus, charge transport properties improve due to the inherent dimeric structure of the LZO in its crystalline form. While *J*_sc_ increases, *V*_oc_ remains constant for almost the total period of stability measurements. The improvement of all the photovoltaic parameters for PEDOT:PSS:CNT devices indicates that the PEDOT:PSS:CNT buffer layer can prevent the diffusion of oxygen or vapor to the photoactive layer (P3HT:ICBA). The reproducibility of the described degradation trends was verified by investigating several identical devices. The corresponding EQE spectra are in agreement with the *J*-*V* characteristics (Additional file 2: Figure S1).

In spite of the moderate efficiencies demonstrated by the present devices compared to the highest state-of-the-art OPVs, their long-term durability and reliability in air is an interesting feature that should be further investigated. Based on our studies, the introduction of the LZO and PEDOT:PSS:CNT buffer layers is the key factor to achieving more stable cells. Thus, the LZO is a very interesting class of compound for the development of stable and efficient OPVs.

The newly synthesized LZO has been investigated as an electron buffer layer in P3HT:ICBA-based inverted OPVs with Al as the top electrode. The performance of the fabricated OPVs has been compared to that of similar devices containing a typical PEDOT:PSS:CNT hole buffer layer between the active layer and Al top electrode. LZO and PEDOT:PSS:CNT cells have higher efficiencies, excellent electron and hole transports properties, efficient electron/hole/exciton blocking properties, better energy alignment at the LZO/ITO interface, and increased cathode work function compared to ITO. In addition, LZO is believed to donate holes/energy to P3HT, which is also beneficial for the device performance.

LZO and PEDOT:PSS:CNT performance was enhanced to up 20%, indicating the most interesting compound for future use. The combination of LZO and PEDOT:PSS:CNT is the most promising for achieving efficient devices. Up to 20% of improvement in PCE was observed with respect to a device containing a GO buffer layer.

The stability of the devices has been accurately studied over 30 days. In spite of the exposure to open air during the whole measurements, the stability and reliability of the OPVs is excellent. In all cases, no significant loss of performance can be observed. The buffer layer effectively blocks the diffusion of oxygen and vapor through the photoactive layers, which has a detrimental effect on the cell stability. Devices containing PEDOT:PSS:CNT are stable, and after 30 days their efficiencies is still close to their initial values.

## Conclusions

The key role of LZO and PEDOT:PSS:CNT in the enhancement of the cell efficiency and stability suggests the use of this class of compounds as an alternative to other widely known buffer materials. The presented results could be applied to more advanced and highly efficient devices as well, combining the advantages of high performance with the long stability. This is ensured by the introduction of LZO and PEDOT:PSS:CNT buffer layers.

## Competing interests

The authors declare that they have no competing interests.

## Authors’ contributions

HPK carried out all electrical measurements; ARBMY designed the study and drafted the manuscript; HMK, HJL, GJS performed UPS, AFM, and refractive index, respectively; and ARBMY and JJ finalized the final manuscript. All authors read and approved the final manuscript.

## Supplementary Material

Additional file 1: Table S1 Environmental degradation parameters of P3HT:ICBA-based devices (2 wt.% of LZO concentration). Click here for file

Additional file 2: Figure S1 Normalized photovoltaic performances of P3HT:ICBA-based devices: (a) J_sc_, (b) V_oc_, (c) FF, (d) PCE for three different LZO concentrations as a function of weeks. Click here for file
