# Supplementary material for: Inverted organic photovoltaic device with a new electron transport layer
Source: Nanoscale Res Lett. 2014 Mar 27;9(1):150. doi: 10.1186/1556-276X-9-150 (PMC3986668; doi:10.1186/1556-276X-9-150)
Supplement: Additional file 1: Table S1 — Environmental degradation parameters of P3HT:ICBA-based devices (2 wt.% of LZO concentration). [file 1556-276X-9-150-S1.doc]

**Table S1.** Environmental degradation parameters of P3HT:ICBA-based devices (2 wt% of LZO

concentration).

| Time | J_sc_ (mA/cm^2^) | V_oc_ (V) | FF (%) | PCE (%) |
| --- | --- | --- | --- | --- |
| Original | -9.35 | 0.86 | 68.58 | 5.49 |
| Week 2 | -8.90 | 0.86 | 68.26 | 5.18 |
| Week 3 | -8.84 | 0.86 | 67.21 | 5.09 |
| Week 4 | -8.55 | 0.85 | 67.17 | 4.89 |
