# Supplementary material for: Inverted organic photovoltaic device with a new electron transport layer
Source: Nanoscale Res Lett. 2014 Mar 27;9(1):150. doi: 10.1186/1556-276X-9-150 (PMC3986668; doi:10.1186/1556-276X-9-150)
Supplement: Additional file 2: Figure S1 — Normalized photovoltaic performances of P3HT:ICBA-based devices: (a) Jsc, (b) Voc, (c) FF, (d) PCE for three different LZO concentrations as a function of weeks. [file 1556-276X-9-150-S2.docx]

**Figure S1.** Normalized photovoltaic performances of P3HT:ICBA-based devices: (a) J_sc_, (b)

V_oc_, (c) FF, (d) PCE for three different LZO concentrations as a function of weeks.
